# Voluntary musical imagery in music practice: contextual meaning, neuroscientific mechanisms and practical applications

**DOI:** 10.3389/fpsyg.2024.1452179

**Published:** 2024-11-11

**Authors:** Chen Meng, Geoff Luck

**Affiliations:** ^1^Department of Music, Art, and Culture Studies, University of Jyväskylä, Jyväskylä, Finland; ^2^Centre of Excellence in Music, Mind, Body and Brain, Department of Music, Art, and Culture Studies, University of Jyväskylä, Jyväskylä, Finland

**Keywords:** music practice, voluntary musical imagery, embodied music cognition, neural activation, functional equivalence, mental practice, practice strategies, PETTLEP model

## Abstract

Practice is acknowledged as a crucial facilitator for musicians to achieve performance excellence. Despite the rich literature on incorporating musical imagery intentionally to improve one’s practice efficacy, limitations remain in the understanding of voluntary musical imagery (VMI) in the context of musical practice. Therefore, our aims in this review are threefold. First, we enriched the interpretation of VMI in the context of music practice through the lens of embodied cognition. Second, we integrated neuroscientific findings to elucidate how the deliberate use of musical imagery parallels physical practice in effectiveness. Third, we synthesize work on the application of VMI in enhancing musical learning from both theoretical and practical perspectives. By providing an integrated overview of voluntary musical imagery, we highlight gaps in the literature and encourage further research on (1) the impact of embodied experiences on VMI formation, (2) optimal imagery content and ratio combination to establish a personalized intervention protocol for more effective musical pedagogy, and (3) on physiological measures to access VMI effectiveness. Additionally, we highlight the crucial implications of VMI for researchers, performers, and music educators.

## 1 Introduction

Musicians, defined as individuals with extended periods of musical training (Zhang et al., 2020), are generally aware that achieving performance excellence requires consistent and extensive effort (Clark et al., 2014; Gregg et al., 2008). Among the diverse ways of improving one’s level of performance, efficient practice strategies have been recognized as crucial in facilitating development of musical expertise (Ericsson et al., 1993; Platz et al., 2014; Sloboda et al., 1996). Since the turn of the millennium, the number of studies concerning the effectiveness of music practice has expanded significantly (How et al., 2022; Miksza, 2011). Researchers from diverse disciplines, including but not limited to music education, performance science, music psychology, and neuroscience, have acknowledged the crucial role of efficient practice strategies in enhancing expertise. Among these strategies, imagery-focused approaches have been identified as particularly beneficial (e.g., Driskell et al., 1994; Ericsson, 1998; Macnamara et al., 2014).

Imagery refers to the inner conscious process of representing or recreating multifaced sensory information (e.g., auditory, visuomotor, etc.) in the mind, particularly in the absence of the external stimulus (Cumming and Williams, 2012; Kosslyn et al., 2001). Grounded upon the nature of imagery as a multisensory cognitive process, research has indicated that individuals are capable of mentally visualizing, hearing, feeling, and rehearsing real-world experiences without actual physical actions (Keller, 2012; Wöllner and Williamon, 2007).

Many researchers have investigated the phenomenon of imagery related to music. One common discovery is that individuals, regardless of their level of musical training, can “hear” melodic tunes in their minds initiated spontaneously (e.g., Liikkanen and Jakubowski, 2020). This phenomenon has been termed involuntary musical imagery (INMI; Williamson and Jilka, 2014) or, more colloquially, an “earworm” (Williams, 2015), in which individuals hear music in their mind’s ear persistently and intrusively without additional effort. Interestingly, however, research has also shown that people are capable of intentionally using musical imagery to mentally rehearse specific music features (e.g., pitch, loudness, and timbre; Cotter, 2019), as well as the physical movement required to create the sound (Bailes, 2009; Godøy, 2022; Holmes, 2005). For example, by interviewing four visually impaired and one fully sighted Western classical pianists about their multifaceted mental experience during music learning and performance, Herbst and Van Zyl (2022) discovered that all participants were able to mentally “feel” the visuospatial chord without the keyboard, “recognize” colors via tonality, and experience elicited emotions during the performance and listening.

While deliberately utilizing musical imagery for performance enhancement has garnered considerable interest within the academic community (e.g., Diana, 2016; Hatfield and Lemyre, 2016; Vilnite and Marnauza, 2023), its interpretation and effect in musical practice varies considerably. To date, most research has been conducted in parallel with individual studies addressing specific aspects of the topic, such as improving memorization (Bernardi et al., 2012), enhancing ensemble coordination (Keller and Appel, 2010), refining performance expressivity (Woody, 2002), and controlling music performance anxiety (Hoffman and Hanrahan, 2012). As a result, a number of issues remain, such as the terminology of musicians’ deliberate imagery usage and the relationship between imagined and actual execution. Additionally, the theoretical frameworks and practical efficacy of voluntary imagery usage in musicians’ practice have yet to be comprehensively synthesized (How et al., 2022).

Here, we explore the existing literature to compare and contrast the theoretical exploration and empirical examination of the deliberate usage of musical imagery within the fields of music education, cognitive neuroscience, and psychology studies. Furthermore, we aim to enrich the understanding of voluntary musical imagery in the context of music practice, clarify the neuroscientific mechanisms behind the benefits of deliberate musical imagery in music practice, and synthesize studies on the intentional use of musical imagery to enhance musical learning from both theoretical and practical perspectives. In so doing, we uncover gaps in knowledge and areas for future empirical development and offer insights into imagery guidance for researchers, performers, and music educators.

## 2 Contextual understanding of voluntary musical imagery

### 2.1 Musical imagery

Recognized as a complex, multifaceted phenomenon, musical imagery has evolved significantly in scientific examination over the past century (Clark et al., 2012). In early studies, musical imagery was viewed as an inner auditory capacity (Seashore, 1919), via which talented musicians could mentally represent and utilize tones and chords (Hubbard and Stoeckig, 1988) or imagine and reproduce musical sounds and sequences (Janata and Paroo, 2006). In the 1970s, Edwin Gordon introduced the concept of ‘audiation’ to describe this process of mentally recalling or comprehending previous musical-sounding experiences. He emphasized this cognitive skill as a prominent tool for music learning and pedagogy (Gordon, 1999, 2021). Later scholars from the music performance field enriched this auditory-only perspective, proposing that musical imagery is not merely a “tune in the brain” but comprises multimodal characteristics of musical performance (Bailes, 2006, 2007a), such as mentally experiencing the movement required to produce the musical sound (Bernardi et al., 2013), seeing music notations in the mind’s eye (Mongelli et al., 2017), and mentally predicting other performer’s action in musical ensemble cooperation (Keller and Koch, 2008). Grounded upon previous foundational exploration, Keller (2012) has defined musical imagery as:

“a multimodal process by which an individual generates the mental experience of auditory features of musical sounds, and/or visual, proprioceptive, kinesthetic, and tactile properties of music-related movements, that are not (or not yet) necessarily present in the physical world” (p. 206).

Given the high frequency of musical imagery experience among student musicians (Bailes, 2007a), its activation and interpretation in the context of music practice has received increasing scientific interest.

### 2.2 Involuntary musical imagery and voluntary musical imagery

In terms of typologies of musical imagery, most studies have focused on two significant dimensions (Cotter, 2019; Williams, 2015): involuntary musical imagery (INMI) and voluntary musical imagery (VMI). A handful of studies have considered a third dimension, described variously as “anticipatory imagery” (Keller, 2012; Liikkanen, 2012) or “involuntary but necessary corollaries of musical activity” (Bailes, 2006, p. 174), both referring to the evoked musical consciousness during performance or musical analysis (Bailes, 2006, 2007a, 2009). Given the dominance of INMI and VMI in the literature, it is these two dimensions we focus on in this article.

INMI is proposed as a mental phenomenon in which music spontaneously enters one’s conscious awareness repeatedly without any effort to create or maintain it (Liikkanen and Jakubowski, 2020; Müllensiefen et al., 2014; Williamson et al., 2012). Early studies of how INMI affects skilled musicians can be traced back to Agnew (1922), who examined autobiographic evidence from 10 renowned composers (including Mozart, Schubert, Tchaikovsky, and Robert Schumann). Agnew argued that, although highly individual characteristics of musical thoughts could be distinguished between each composer, the compositional ideas that came to their mind were commonly in the form of INMI (Agnew, 1922).

By employing experience-sampling forms to record student musicians’ inner musical consciousness via phone prompts six times a day for a week, Bailes (2006, 2007a) empirically demonstrated the high frequency of INMI occurrence in music students’ daily routines. Similar results have been demonstrated by Beaty et al. (2013) and Hyman et al. (2013), who further suggest that music students report experiencing INMI more frequently than their non-musical counterparts, indicating a correlation between musical training and the occurrence of INMI. However, the evidence is not uniformly consistent across all studies. Both Liikkanen (2012) and Williamson and Jilka (2014) found that individuals with higher music expertise appear to have a lower frequency of involuntary musical imagery. To further complicate matters, Beaman and Williams (2010) and Müllensiefen et al. (2014) did not find a significant relationship between INMI frequency and musicianship, while Floridou et al. (2015) and McCullough Campbell and Hellmuth Margulis (2015) reported mixed or negative results regarding the impact of musical training on INMI susceptibility. Recently, Liikkanen and Jakubowski (2020) conducted a comprehensive review of the empirical literature related to INMI from multiple viewpoints, including its maintenance, activation, personal differences, and content of representation. Despite INMI being well-evidenced as an essential cognitive component in everyday life circumstances, its influence and causal efficacy in music practice remain to be further explored.

### 2.3 Meaning of voluntary musical imagery

In contrast to INMI, some researchers have proposed that certain individuals, notably those with musical training, possess a higher capacity to consciously evoke and manipulate musical details without the presence of an external stimulus (e.g., Gregg et al., 2008; Holmes, 2005). For example, a study by Aleman et al. (2000) demonstrated that individuals with over 5 years of musical training possess enhanced musical imagery abilities, enabling them to outperform those with more limited musical training (i.e., less than 2 years) in tasks requiring the mental manipulation of musical and non-musical sounds. Further auditory research has identified specific aspects of musical imagery that skilled musicians can adeptly control mentally. These include reliving and adjusting the musical pitch (Janata and Paroo, 2006), loudness (Bishop et al., 2013), and instrumental timbre (Bailes, 2007b; Crowder, 1989; Halpern et al., 2004). With these capacities, individuals with greater levels of musical training are able to mentally rehearse their repertoire thoroughly before going on stage (Clark and Williamon, 2011; Freymuth, 2004) and mentally practice the music without touching the instrument (e.g., Davidson-Kelly et al., 2015). While the phenomenon of actively representing musical experiences mentally, in the absence of direct stimuli, is widely recognized, the terminology used to describe this process is not consistent across studies, being referred to variously as “voluntary musical imagery,” “volitional musical imagery,” or broadly as “musical imagery.” This inconsistency in terminology leads to confusion about what each term specifically means, complicating efforts to synthesize and compare findings across different studies. Additionally, the specific interpretation of this concept in music practice remains unclear.

For instance, Williams (2015) defined VMI as the “musical experience that was called to mind through the active choice of the person in the absence of direct sensory instigation of that experience” (p. 5), emphasizing the subjective intentionality of musical imagery when musicians employ it for their mental practice or composition. More recently, Godøy (2019, 2022) integrated music theory, phenomenology, and cognitive science to formulate the deliberate initiation and maintenance of musical images in minds as *volitional* musical imagery. He argues that the intentional control or recreation of musical sound in the mind is triggered by the motions of sound production. This conceptualization emphasizes the role of motor cognition in engagement of musical imagery, where musicians can mentally hear or compose the music by visualizing the performing movements (for more detail, see Küssner et al., 2022).

While Williams (2015) and Godøy (2019, 2022) provide valuable insights into understanding VMI, their interpretations have limitations. Specifically, although Williams highlights the conscious decision to recall musical imagery without sensory input, her definition may overlook the fact that the “music experience” for musicians is derived intensively from the physical sensations and movements during their extensive daily practice. By focusing solely on mental choice, Williams’ definition does not fully account for the role of embodied experience in forming and sustaining vivid musical imagery, such as the tactile feedback from an instrument, muscle memory developed through repetitive practice, and kinesthetic sensations of playing or singing (Steenstrup et al., 2021). This neglect of embodied aspects risks oversimplifying the richness of musicians’ imagery content. Similarly, while Godøy highlights volitional musical imagery as the dynamic interplay between motion imagery and auditory imagery, the focus on motor imagery alone may oversimplify the multi-sensory musical experience integral to musical performance and appreciation. As such, a gap exists that emphasizes the need for an interpretation of VMI that aligns with the unique requirements of musicians.

To deepen our understanding of the deliberate use of musical imagery in the music context, we consider it as *voluntary musical imagery* and propose a more comprehensive interpretation of musical imagery from the perspective of embodied music cognition in the context of music practice. That is, VMI is not merely an intentional mental representation or recreation of the musical performing experience but also an embodied phenomenon in which cognitive and physical processes are deeply interwoven.

This interpretation of VMI aligns with the foundational principles of embodiment studies. Embodied research indicates that cognitive processes like thinking, perceiving, and understanding are formed by corporal interaction with the real-world environment (Varela et al., 1991; Varela F. J. et al., 2016; Leman and Maes, 2014). In the music context, Leman et al. (2018) describe embodied music cognition as the “cognition in perception” (Leman et al., 2018, p. 748) – that cognitive processing of music, such as learning, recalling, and predicting, are formed by the interaction of our body with music (Dahl et al., 2023; Hashim et al., 2023). From this perspective, music imagery is integrally linked to the perception and bodily experiences of engagement with music (Leman, 2008; Niedenthal et al., 2005; Thompson and Luck, 2012). Music practice exemplifies this embodied perspective (Cox, 2016). For instance, Gebel et al. (2013) investigated the neural auditory-motor loop between trumpeters and pianists with long-term (c. 15 years) training. They found that the degree of auditory to motor cortical activation varied between the two groups of instrumentalists, and the cause of this cognitive processing difference was influenced by the physical and sensory systems developed through their context-specific training. Similarly, Kajihara et al. (2013) examined the influence of musical expertise and pedagogical approaches on auditory-motor coupling. By asking non-musicians, conservatory-trained violinists, and pupils trained with either traditional and Suzuki methods to match pitches with mental finger-numbers, their study illustrated that experienced violinists and learners trained under the Suzuki method demonstrated significant pitch-to-finger mapping accuracy and speed. Their result suggests that the musical auditory-motor coupling ability is not innate but could be shaped by specific embodied pedagogy. These findings reinforce the idea that embodied training experiences profoundly shape skilled musicians’ mental representation (Godøy, 2003).

Wilson (2002) divided embodiment into “online” and “offline” forms. Specifically, the “online” form indicates perceptual activities and cognitive processes that are directly tied to real-world interactions (Leman and Maes, 2014; Niedenthal et al., 2005), such as expressive musical intentions through body gestures (Thompson and Luck, 2012). On the other hand, “offline” embodiment refers to cognitive activities that occur without direct engagement with the environment, such as practice without auditory feedback. From this viewpoint, Jäncke (2012) employed scalp EEG and standardized low-resolution electrical tomography (sLORETA) to examine the dynamics of the auditory-motor system in piano performance. By examining six professional pianists’ performance with and without auditory feedback, they revealed a causative influence from the auditory cortex (AC) to the premotor cortex (PMC). This influence was found to persist significantly in the non-auditory condition, as well as during rest state. These findings revealed that pianists can activate these auditory-motor pathways even in the absence of physical performance. Recently, Klein et al. (2016) used high-density electroencephalography to evaluate whole-brain functional connectivity during resting states in both string players and non-musicians. Their results showed that similar brain regions activated during musical performance remain functionally connected even at rest. These studies demonstrate that intentional mental imagery has the capacity to embody real-world perception and re-experience it mentally without physical involvement.

As such, the understanding of VMI was enriched from two complementary perspectives: the embodiment-imagery and the imagery-embodiment directions. Specifically, the “embodiment-imagery” perspective illustrates the function of VMI as an “offline” simulation of multimodal real-world cognition (Bailes, 2019; Niedenthal et al., 2005). For example, musicians often report physically “feeling” the music even when they are merely imagining it, indicating that VMI involves simulating the physical actions associated with music production (Godøy and Jørgensen, 2001). This engagement suggests that music learners may enhance their understanding and execution of musical pieces by involving not only auditory representations but also motor executions during mental practice (Schiavio et al., 2014). Conversely, the “imagery-embodiment” direction offers insights into how VMI can facilitate physical music practice. By intentionally engaging in VMI, musicians can mentally rehearse their performances, which may reinforce the motor and auditory pathways involved in actual performance. To further advance our comprehension of VMI, we review its neuroscientific exploration and practical application in musicians’ practice routines below.

## 3 Neuroscientific mechanisms of voluntary musical imagery

Studies of VMI have garnered significant interest among neurocognitive scientists perhaps due to its features of prevalence in musicians’ mental practice and vividness in musical details (Zatorre, 2003; Zatorre and Halpern, 2005). Along with the evolution of neuroscientific technologies, researchers are able to deepen our understanding of VMI beyond behavioral and self-report experiments (Levitin and Grafton, 2016) via a range of experimental tools, such as magnetoencephalography (MEG), electroencephalography (EEG), positron emission tomography (PET), and functional magnetic resonance imaging (fMRI). By employing such technologies, scholars have made valuable contributions to our understanding of VMI in terms of neural activation (e.g., Zatorre et al., 1996), differences compared to music perception (e.g., Herholz et al., 2012) and network correlations in the brain (e.g., Zatorre and Halpern, 2005).

### 3.1 Neural activation during VMI

Early studies by Zatorre and Halpern (1993) demonstrated that musical imagery activates neural regions involved in auditory perception. Their research on patients with temporal-lobe excisions showed that imagining familiar tunes engages the same neural areas as actually hearing them. Later studies confirmed these results, with findings indicating that the auditory cortex is similarly activated during both mental imagery of music and actual music listening, even without external sound stimuli (Zatorre et al., 1996; Zatorre and Halpern, 2005).

Previous fMRI studies of mental music evaluation tasks, such as pitch judgment (Zatorre et al., 1996), melodic extensions (Halpern, 1999), timbre recognition (Halpern et al., 2004) and tonal accuracy of imagined melodies (Herholz et al., 2008), have revealed that the secondary auditory cortex and the auditory association areas are largely activated and similarly overlapped between auditory imagery and auditory perception (Kosslyn et al., 2001). More recently, Regev et al. (2021) conducted a mapping study comparing the neural response between imagined and perceived music. fMRI scans of 25 participants instructed to deliberately imagine pre-memorized music extracts under tapping, no tapping and listening-only conditions revealed that imagery of musical melodies consistently activated participants’ early and associative auditory cortices. Specifically, the bilateral middle superior temporal plane (mSTP), areas overlapping with early auditory processing loci in Heschl’s gyri, anterior and posterior STP regions, and lateral parts of the right superior temporal gyrus (STG) all reflect the sensory regions activated during actual melody perception. These commonalities of the activation in brain regions reflect the equivalent neural function between VMI and the perception of music (Halpern and Overy, 2019; Levitin and Grafton, 2016).

Neuroscientific studies have identified parallels in brain activation between executing and imagining music performance movements (see Lotze, 2013, for a review). For example, by analyzing fMRI data collected from 12 pianists performing Bartok’s Triolak in a right-hand-only task under live and imagery conditions, Meister et al. (2004) reported that bilateral parietal cortical activations in the supplemental motor area (SMA), premotor areas, the precuneus, and the medial part of Brodmann Area 40 had significant overlap in the two conditions. These findings highlight the rationale of action simulation, where motor imagery functions in an analogous neural mode with motor execution when voluntarily imagining musical movement (Keller, 2012; Lotze, 2013). Later studies found that the right superior parietal lobule (SPL) was highly engaged when performers reported mentally “seeing” their finger action while playing their instrument (Bastepe-Gray et al., 2020; Lotze et al., 2003; Tanaka and Kirino, 2017). Moreover, the SMA, pre-SMA, and premotor cortex (PMC) were found to be actively engaged and particularly crucial for the coordination of imagined performance (Gelding et al., 2019; Halpern, 2001; Halpern et al., 2004; Tanaka and Kirino, 2017; also see Lima et al., 2016 for a review), especially due to their functions of movement generation, control, preparation, anticipation and sequencing arrangements (Tanaka and Kirino, 2017). This overlap of brain regions in both motor execution and imagery implies functionally equivalent possibilities for musicians, where voluntary imagery might reinforce physical practice by engaging the same neural circuits, potentially offering complementary benefits (Driskell et al., 1994).

### 3.2 Neural correlates of VMI

Recent neuroscience research has revealed that diverse brain networks are activated during conscious musical imagery (Bastepe-Gray et al., 2020; Halpern, 1999; Lotze, 2013; Lotze et al., 2003; Zatorre and Halpern, 2005). For example, Herholz et al. (2012) conducted fMRI examinations on 10 participants ranging from non-musicians to professionals to compare their neural activity while imagining and perceiving familiar tunes with lyrics. Regardless of their music expertise, an extended network, including the prefrontal cortex, supplementary motor area (SMA), intraparietal sulcus, and cerebellum, was activated during such imagery tasks.

Musicians’ engagement with musical imagery has also been explored from a neuroscientific perspective. For instance, Langheim (2002) employed fMRI scans to demonstrate that a network involving the prefrontal cortex, parietal cortex, and lateral cerebellum was activated collectively in string musicians when engaging in intentional musical imagery. This discovery highlights VMI’s capacity to orchestrate the complex spatial and temporal aspects of musical performance, suggesting its critical role in enhancing musical execution through mental practice. Similar research has been conducted with vocalists. For instance, a study by Kleber et al. (2007) with 16 expert classical vocalists revealed that a correlated network encompassing frontal and parietal regions was activated during VMI. Moreover, Kleber et al. (2007) reported that professional singers employed different brain systems for imagined versus overt singing of Italian arias, with imagery involving a broader network of higher-order associative functions and more intense activation in the prefrontal and limbic areas. In light of these findings, it is evident that VMI activates diverse networks in the brain. Moreover, the identification of specific regions activated during imagery tasks further elucidates VMI’s potential mechanisms in supporting functions such as memory retrieval, tonal working memory activation, and mental monitoring (Ding et al., 2019; Herholz et al., 2012; Hubbard, 2010).

Notably, Bastepe-Gray et al. (2020) discovered that the breadth of these networks and the depth of their activation during VMI varies depending on the method of mental representation employed. To investigate this, they conducted fMRI scans on seven oud players who employed three different mental practice strategies: eyes closed, eyes open, and following a musical score, with an eyes-open rest serving as a control. Their findings indicate that, while patterns of activation during VMI are consistent with prior neuroscientific research, the level of cortical activation is influenced by the chosen mental practice strategy. For example, despite all mental rehearsal strategies enhancing internal connectivity of the occipital lobe bilaterally, the strategy of vivid imagery with closed eyes resulted in the most widespread activation in these lobes. Given that different imagery models can trigger sensory and motor neural areas to differing extents, these results underscore the importance of designing context-specific VMI guidance based on a thorough understanding of the brain networks involved (Bastepe-Gray et al., 2020).

### 3.3 Differences between VMI and music perception

While neuroscientists have explored the overlapping mechanism of imagined and actual perceived music, their inherent differences have also been highlighted. Studies have revealed that the primary auditory cortical regions have limited activation when recalling music consciously compared to music listening and are only engaged when participants imagine familiar songs with no lyrics (Kraemer et al., 2005; Levitin and Grafton, 2016; Regev et al., 2021). Similarly, the primary motor cortex is found to be less involved during imagery than in the actual movement execution (Lotze et al., 1999; Lotze et al., 2003). These different areas of brain activation, between imagining and actually perceiving music or executing movements, highlight differences in cortical processing of internal versus external stimuli.

Additional differences in the sequence of neural activations between listening and imagining music have been proposed. Kosslyn et al. (2001) have stated that “mental imagery occurs when perceptual information is accessed from memory…perception occurs when information is registered directly from the senses” (p. 635). Recent findings by Ding et al. (2019) provide additional evidence supporting differentiated cortical activity patterns during music perception and recall. By analyzing Electrocorticography (ECoG) data from 10 epilepsy patients, their study demonstrated distinct temporal sequences in neural activation. Specifically, during music listening, the initiation of cortical activity progresses from the sensory cortex to the frontal cortex, exemplifying a bottom-up approach. In contrast, music recall involves a reversed sequence, where activation begins in the frontal cortex and moves toward the sensory cortex, reflecting a top-down process. These finding contribute to our understanding of the directional dynamics of neural processing in auditory tasks. Additionally, they reveal that the unique induction mechanism of musical imagery, as a cognitive process, is able to retrieve long-term memory stored previously or reactivate the sensory regions of the brain (Halpern, 1999). Meanwhile, the participation of the frontal cortex also implies that imagining music voluntarily is a higher-level cognitive neural activity that involves multiple regions of the brain (Bastepe-Gray et al., 2020; Centanni et al., 2020).

In summary, extensive research on VMI has uncovered an intricate neural mechanism underpinning musical imagery and perception, delineating the dynamic interplay among cognitive processes involved. Through the utilization of sophisticated neuroimaging techniques, scholars have elucidated the parallel cortical activation contents during music perception and VMI, discovered their constitutional differences, and underscored the critical role of the auditory and motor cortex in a musician’s imagery process. This equivalence in neural functioning provides a solid foundation for the use of VMI during mental practice, which might be integrated into physical practice routines as a personalized protocol, offering musicians an adaptable approach to their unique needs in skill acquisition and performance enhancement.

## 4 Applications of VMI: theoretical and practice perspectives

### 4.1 Voluntary musical imagery in musical practice: theory comparison

Building upon the neuroscientific findings concerning the cortical overlap between imagined and actual practice execution, scholars from music psychology and pedagogy have discovered that expert performers, through the mental representation of specific performance details, can facilitate the planning and evaluation of potential actions and enable precise monitoring of and feedback for their performance (e.g., Ericsson, 1998). The theoretical frameworks that utilize VMI in achieving music improvement have focused on two pivotal theories: deliberate practice (DP) and self-regulation learning (SRL).

#### 4.1.1 Deliberate practice theory

Music practice is historically viewed as a structured and deliberate learning process aimed at enhancing musical abilities. Such perspectives are rooted in the seminal exploration of Ericsson et al. (1993). Derived through qualitative and quantitative analysis of the development of selected conservatory violinists, Ericsson and colleagues discovered the acquisition of expert-level excellence was associated with early-start, time-consuming, daily deliberate practice. Unlike general physical training, deliberate practice was defined as knowledge-driven, goal-oriented, and improvement-focused exercises (Ericsson, 1996, 1998; Ericsson and Harwell, 2019; Passarotto et al., 2022). This influential work shaped understanding of how expert-level skills in music are achieved not merely through hours of practice but through engaging in specifically-designed exercises (Mazur and Laguna, 2019; Sloboda et al., 1996). In a later meta-analysis, Platz et al. (2014) identified a strong correlation [r_c_ = 0.61; 95% CI (0.54, 0.67)] between performance improvement and the quality and quantity of practice, further reinforcing the importance of deliberate and effortful practice inputs. More recently, Passarotto et al. (2022) developed the Deliberate Practice in Music Inventory (DPMI), a novel tool that measures the quality of practice across various musical genres and expertise levels quantitatively. The positive results obtained from the DPMI test confirm the broad effectiveness of deliberate practice in substantially enhancing musical expertise.

**Figure 1:**
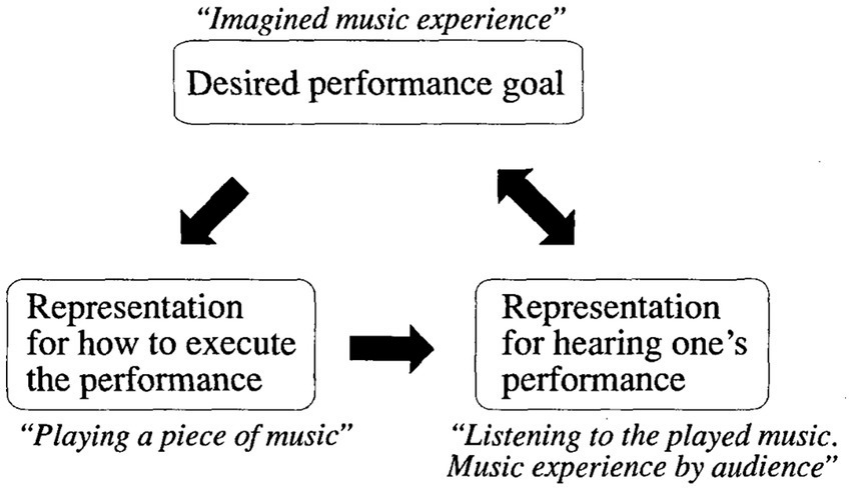
Imagery-perception loop mediates expert music performance. Adapted from (Ericsson, 1998).

Individual differences, such as innate capacity or talent (Ericsson and Harwell, 2019; Macnamara et al., 2014), as well as the vividness of mental representation and the ability to manipulate imagery content (Highben and Palmer, 2004), have been argued to contribute to the acquisition of expertise. However, to advance oneself as a self-improving artist, voluntarily imagining the desired performance in vivid detail and aligning it with performance action has been commonly agreed upon as a necessary element of deliberate practice (Ericsson, 1998; Ericsson and Harwell, 2019; Lehmann and Ericsson, 1997). As such, Ericsson (1998) presented a VMI application model that included three specific roles of musical imagery in representing performance (see Figure 1): (a) the imagery of the desired musical sound as the performance goal, (b) the imagery of the motor performance executions, (c) the imagery of the evaluation in terms of the performance (also see Lehmann and Ericsson, 1997).

Specifically, the first imagery is the ideal auditory image of the music, representing the performance goal that musicians want to achieve in their practice sessions. The second representation involves motor imagery, focusing on the physical action needed to execute the instrument and meet the performance goals. The third representation entails the real-time assessment of the musician’s current performance compared to the intended goal.

Within this process, VMI is integral to forming the mental content of deliberate practice. It enables both student and expert musicians to mentally simulate musical pieces without external stimuli; musicians thus can ‘hear’ music in the absence of sound (Brodsky et al., 2003; Lotze, 2013). However, the efficacy of this practice depends to a large degree on the vividness with which an individual can imagine the music (Lotze, 2013; Aleman et al., 2000). Moreover, VMI facilitates the mental rehearsal of physical actions necessary for performance, strengthening the engaged neural pathways associated with these movements. This process not only aids in the biological planning of movement sequences but also reduces the physical exertion required in actual performances, thereby enhancing overall musical execution (Keller and Appel, 2010). In comparison to physical practice alone, the application of VMI enables musicians to visualize the expected performance and monitor their ongoing performance mentally (Ericsson, 1998).

#### 4.1.2 Self-regulation learning theory

For decades, self-regulation learning (SRL) theory has been extensively studied and validated by researchers as an effective framework for facilitating musical learning (Leon-Guerrero, 2008; Nielsen, 2001; Schunk and Zimmerman, 2009; Varela et al., 2016). Initially, the concept of SRL was constructed into three key components from a social-cognitive perspective. These include the strategic selection of learning processes, the implementation of strategies aimed at achieving academic goals based on self-efficacy beliefs, and a dedication to academic aspirations (Zimmerman, 1989, 1990).

**Figure 2:**
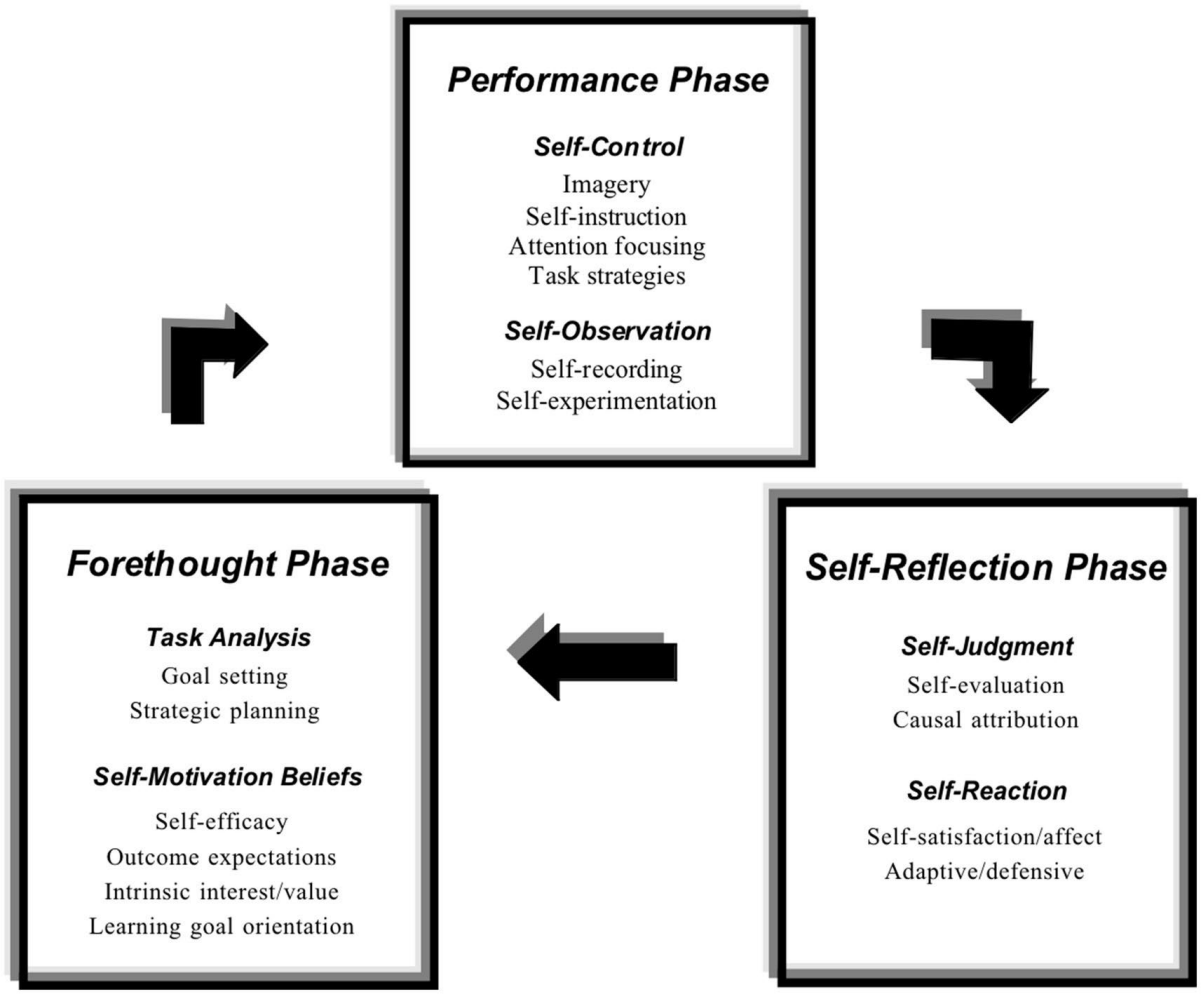
Self-Regulation Process. Adapted from (Zimmerman, 2002).

The early framework of SRL portrayed it as a deliberate, intentional act of acquiring knowledge or skills. This process involves a triadic relationship between the individual, their behavior, and the environment (see Zimmerman, 1989, for more details). Researchers have found that self-regulated learners not only employ various strategies but also understand how these strategies impact their learning outcomes. This understanding underscores that SRL involves a dynamic interaction between cognitive, emotional, and physical elements (Zimmerman, 1990, 2000). Contemporary views of SRL describe it not just as a learning technique, but as a cyclical model of mental self-regulation that supports lifelong learning. This model comprises three phases: the forethought phase, which involves task setting and motivational arousal; the performance phase, which focuses on individual control and ongoing self-monitoring; and the self-reflection phase, which includes evaluations and emotional assessments of the learning process (Panadero, 2017; Zimmerman, 2000). Within these phases, the use of voluntary imagery plays a crucial role in self-control during the performance phase (Figure 2).

Zimmerman (2002) describes the application of imagery as a mnemonic strategy. This technique involves mentally linking new knowledge with previous understanding. For instance, when studying the Spanish word “pan” as “bread,” a student may create an image of “bread pan” to enhance comprehension. In this condition, imagery serves as a tool to bridge new information with familiar image concepts, which could positively impact the learning process.

In the context of music practice, Renwick and McPherson (2002) discovered that a clarinetist, while engaging in the SRL cycle to master new pieces, voluntarily visualized her tutor’s performance as a reference to assess her own pitch accuracy during practice. Via an analysis of over 300 surveys from music grade exam instrumental candidates, McCormick and McPherson (2003) suggested that using musical imagery as part of SRL can enhance self-efficacy, which in turn, is likely to improve performance quality. In a related study, Clark and Williamon (2011) conducted a nine-week mental skills training program for conservatory-level students, based on the SRL framework. The program included techniques in mental rehearsal, performance preparation, goal-setting, relaxation, motivation, and arousal control. The students who underwent this training showed notable improvements in self-efficacy, self-awareness, and practice duration compared to those in the control group. Moreover, students who engaged in SRL training reported more frequent and vivid use of imagery in their practice routine and rehearsal (Hatfield, 2016). While these findings underscore the benefits of incorporating intentional musical imagery into SRL, how imagery interacts with the components of SRL and to what extent these improvements are solely attributable to the use of VMI is still unclear.

In essence, while the deliberate use of musical imagery in self-regulated practice is only one part of the broader SRL cycle, its importance cannot be underestimated. As a cognitive strategy, it enhances the integration of new information with existing knowledge. Furthermore, it plays a critical role in managing focus and emotional states, which are crucial for effective and efficient practice (López-Íñiguez and McPherson, 2024; McPherson et al., 2017). Given its significance, future research should focus on optimizing the use of imagery specifically within music practice, leading to more nuanced and effective SRL methodologies tailored to the unique demands of music learners.

#### 4.1.3 Comparative discussion

Despite the theories of DP and SRL being embedded in distinct conceptual grounds, three commonalities are apparent when applied to the context of music practice, particularly in relation to VMI. First, both theoretical models highlight the critical role of VMI in enhancing musicianship by requiring musicians to mentally rehearse complex compositions, improving their execution and deepening their interpretative skills. Second, the emphasis on task-setting and goal-oriented exercises within DP resonates with the strategic execution of learning strategies in SRL. The significance of deliberately employing musical imagery to present or sustain the desired goal/task vividly to assist skill acquisition is highlighted in both (Cotter, 2019). Third, both theories recognize the value of self-monitoring and regulation in the learning process, where the use of VMI enables musicians to assess their performance against desired outcomes, thereby enabling adjustments and improvements.

Despite these commonalities, each theory differs in its primary focus and application. For example, DP is centered around the accumulation of expertise through extensive, effortful practice, with VMI serving as a tool to present and anticipate the performance specifics. On the other hand, SRL presents a more comprehensive learning model that incorporates cognitive, behavioral, and contextual elements. Within this model, VMI is particularly associated with enhancing memorization and regulating emotions. As both theories share a similar commitment to fostering long-term autonomous learning ability (Ericsson, 1998; Zimmerman, 2002), further longitudinal studies illustrating the effects and efficacy regarding musical expertise development in the DP and SRL approaches, especially the role of VMI over the long term, would be of great benefit.

### 4.2 Voluntary musical imagery in mental practice

#### 4.2.1 Effect of VMI on mental practice

Mental practice (MP) has been well recognized as a means of enhancement that offers extensive benefits for performers in fields like sports (Maring, 1990), music (Coffman, 1990), and surgery (Conlin et al., 2016). In music research, MP refers to the training strategy in which musicians mentally “create or recreate an experience that is similar to a given physical event” (Connolly and Williamon, 2012, p. 224) in the absence of overt muscular motions (Driskell et al., 1994; Highben and Palmer, 2004). This technique has been proven to benefit musicians by enhancing learning efficiency (Coffman, 1990; Lim and Lippman, 1991), increasing practice motivation (Clark and Williamon, 2011), and reducing performance anxiety (Hoffman and Hanrahan, 2012). When engaging in MP, the application of VMI proves crucial. This necessity arises because MP tasks require participants to visualize musical notes and their execution in the mind, a process fundamentally reliant on VMI (e.g., Ross, 1985; Bernardi et al., 2012).

For instance, early research by Rubin-Rabson (1941) identified the beneficial effects of combining mental practice with physical training on music memorization. In this study, participants were tasked with a mental practice section of the piano notes and kept the mental image as vivid as possible. Subsequent research further investigated whether specific imagery content may have an effect on mental practice efficacy. Highben and Palmer (2004) conducted studies on aural and finger sequence discrimination with pianists to assess the impacts of auditory and motor imagery. Their data indicated that, despite individual differences in imagery ability, the auditory aspect of VMI is more crucial for ensuring music memory. Expanding on previous research, Brown and Palmer (2013) studied how VMI influences formally trained pianists’ encoding and retrieval of novel melodies. They found that both auditory and motor imagery improve the recall of pitch accuracy. However, a well-developed auditory imagery capability is particularly effective at enhancing musicians’ accuracy in pitch sequence learning and ensuring consistent temporal control during performance recall. This suggests that auditory imagery should be a primary focus in the development of musical imagery memorization strategies. Moreover, Brown and Palmer (2013) noted that advanced pianists benefit significantly from employing a feed-forward approach using auditory imagery. This strategy involves anticipating and mentally rehearsing the motor sequences necessary for performance, rather than relying solely on feedback from actual auditory inputs during the performance. This proactive use of VMI allows musicians to better prepare and execute their performances (Bernardi et al., 2013; Keller, 2012).

Comparative studies of solely physical practice (PP), MP only, or a combination of both have further highlighted the practical efficacy of VMI. These studies indicate that although PP is the most effective method for learning and memorization, integrating MP into PP can yield comparable effectiveness to PP alone while offering additional advantages. Evidence has suggested that by mentally rehearsing music, trombone students can develop a deeper understanding and more accurate anticipation of the works they are studying (Ross, 1985). This combination approach also enhances the retention of musical notation and performance details (Iorio et al., 2022). Furthermore, incorporating MP into PP optimizes musicians’ practice time and reduces their necessity for extensive physical rehearsal, thereby reducing the risks associated with over-practicing (Bernardi et al., 2012). Additionally, MP enhanced with auditory input, such as background recording (Lim and Lippman, 1991) or verbal singing (Steenstrup et al., 2021), is more effective than MP alone, and dividing imagery content into segments or starting with simple tasks may provide more productive benefits for the advancement of mental practice (Cahn, 2008). It’s worth pointing out that, across all skill levels (i.e., professional and amateur), musicians who engage in *some* form of practice prove superior to those who do not practice at all (i.e., PP only ≥ MP + PP > MP with recording > MP only > no practice; Ross, 1985; Coffman, 1990; Highben and Palmer, 2004; Bernardi et al., 2012).

Beyond the advancement in music learning, memorizing, and skill acquisition, researchers have shown that employing VMI in music practice offers significant benefits in psychological aspects, including improving focus, minimizing distractions, and enhancing mental toughness (Gregg et al., 2008).

#### 4.2.2 Voluntary musical imagery strategies of mental practice

##### 4.2.2.1 The NBO method

Recently, Davidson-Kelly et al. (2015) conducted a longitudinal study exploring the effects of Nelly Ben-Or’s (NBO) deliberate musical imagery strategy on pianists’ memorization and skill acquisition. Unlike the previous approach of integrating mental practice after physical practice had been established (Rubin-Rabson, 1941), NBO emphasized the early use of VMI to construct a comprehensive performance. This included mentally imagining the music scores, sounds, keyboard layouts, and finger positions before physically engaging with the instrument. Significant findings emerged from this method. Participants reported that the NBO method offered greater clarity and connection with the music by sharpening their focus on the sound and the instrument, improved their learning efficiency, and reduced physical tension. Interestingly, physical practice was not the participants’ top choice (this time ranking seventh out of 12 strategies). In contrast, imagery of the keyboard became the participants’ most favored approach, which represents a notable shift from Highben and Palmer’s (2004) emphasis on auditory imagery. Davidson-Kelly et al. (2015) explained this transfer as being caused by the specific bodily-focused feature of the NBO method. In some way, their different outcome implies the plasticity of a VMI intervention in influencing the different aspects of music learning and enhancement. However, the study also highlighted challenges. The effectiveness of VMI was influenced by the pianists’ existing inner imagery capabilities and their experience with mental practice. Consequently, while the NBO method has shown that VMI can improve musicians’ practice efficiency and have the plasticity to adapt to different needs, further research is required to develop VMI usage guidelines that facilitate musicians’ physical training more adaptively and effectively.

##### 4.2.2.2 The PETTLEP model

The interdisciplinary approach of incorporating sports imagery strategies into music practices has received a great deal of attention in recent years (Gregg et al., 2008; Hays, 2012; Pecen et al., 2016). Wright et al. (2014) discussed the possibility of applying the PETTLEP (Physical, Environment, Task, Timing, Learning, Emotion, and Perspective) model as a VMI framework for musicians to improve performance. Developed by Holmes and Collins (2001), the PETTLEP model serves as an intervention guideline instructing athletes’ mental practice, in which each component is designed to activate the correlated neural area that provides similar effects to the actual movement. Based on the commonalities with imagery tasks in providing efficiency for both musicians and athletes (Driskell et al., 1994), Wright et al. (2014) argued that each element of the PETTLEP model has a corresponding function in musical imagery practice. For instance, the Physical element refers to the deliberate imaging of the performing movements with/without holding instruments; the Environment element indicates the mental imagery of performing in the musical venue; and the Perspective element requires musicians to evaluate their performance via imagery from a first-person or third-person perspective. Compared with previous investigations, Wright et al. (2014) suggested specific content for designing musical imagery interventions under the PETLEP framework. However, the efficacy, optimal combination (i.e., select the elements with needs or all-included), and suitability of each element from the PETTLEP has received limited empirical exploration in musical training.

## 5 Discussion

By synthesizing existing research on the embodied phenomenon description, neural mechanisms, and practical applications of voluntary musical imagery in music practice, our review has highlighted crucial implications of VMI for researchers, performers, and music educators. However, we discovered significant gaps in previous research on VMI which highlight the need for further research within three critical areas: (1) the impact of embodied experiences on VMI formation, (2) the optimal imagery content and ratio combination to establish a personalized intervention protocol for more effective musical pedagogy, and (3) the measurement from a physiological perspective.

### 5.1 Exploring the impact of embodied experiences on VMI formation

Upon assessing the definition of VMI from previous literature, we proposed a novel interpretation. This new understanding, situated within an embodied music cognition framework, emphasized the intertwined role of cognition and body in shaping VMI (Godøy and Leman, 2010; Leman and Maes, 2014). However, this perspective also introduces certain limitations that merit further discussion. For instance, while this new interpretation underscores the importance of physical sensations in influencing VMI, direct empirical evidence linking these bodily experiences with musical imagery processes remains scant. This lack of robust data limits our understanding of how physical sensations directly affect the formation and quality of VMI.

To address these gaps, future research should investigate the specific mechanisms through which body sensations interact with musical imagery. One critical question might be: how do different embodied states influence the vividness and accuracy of VMI? For instance, if physical embodiment significantly affects VMI’s application, then inducing a state of physical relaxation (Williamon, 2004) might enhance the vividness and accuracy of musical imagery compared to a state of tension (Holmes and Collins, 2001). Empirical studies could test this prediction by comparing musicians’ VMI usage efficacy under varying physical conditions. Additionally, empirical research could investigate whether musicians’ mastery level, such as physical skills and performance achievement, influences the formation, quality, and sophistication of VMI. This exploration could provide deep insights into the role of embodiment and expertise in shaping VMI across various levels of musical proficiency. For instance, if embodied experience and higher musical achievement significantly impact VMI, we would expect expert musicians with refined techniques and interpretative skills to exhibit more vivid, detailed, and sophisticated VMI than beginners. Investigating this potential bidirectional relationship will deepen our understanding of how VMI and musical expertise influence each other, enhancing practical applications in music education and therapy.

### 5.2 Optimizing content and protocol for VMI effectiveness enhancement

Despite the recognition of VMI in the development of musical expertise and its multimodal nature during musicians’ mental simulations (Küssner et al., 2022; Keller, 2012), a considerable knowledge gap remains. This gap pertains to our understanding of the content and proportions of various type of VMI in musicians’ practice conditions (Cumming and Eaves, 2018). Specifically, there is insufficient research on how the particular content of imagery (i.e., what is mentally represented) and the engagement degree of various sensory categories (i.e., how much each type of imagery is imagined) impact the effectiveness of VMI. Consequently, music educators, psychologists, and performers may lack clear guidelines for optimizing VMI applications to improve musicians’ learning efficacy.

Therefore, further research is imperative to investigate the commonalities in musicians’ VMI content and to determine the potential individualized multi-model imagery framework that enhances music practice. Such research might raise the question of how to integrate auditory, motor, and visual imagery to maximize learning efficiency and performance quality in musicians. For example, assuming an integration of auditory and motor imagery leads to superior performance outcomes in technical precision and expressive depth, musicians who employ both categories equally could expect to show greater improvements compared to those who focus predominantly on utilizing one kind of imagery. This further investigation could yield crucial insights that enable music educators and performers to most effectively tailor VMI applications to specific practice scenarios. For example, strategies that enhance solo performance preparation may not be equally beneficial for ensemble coordination.

Additionally, the effectiveness of these optimized VMI strategies might vary widely due to individual differences in imagery capabilities and extent of musical training, as seen in previous studies (Davidson-Kelly et al., 2015; Williamon, 2004; Holmes and Collins, 2001). This variability underscores the necessity for the development of a comprehensive VMI intervention protocol, akin to the PETTLEP model but tailored for the music domain. Such a protocol would require collaborative efforts from music educators, psychologists, and performance coaches. As such, future exploration is required to offer tailored guidance for musicians, facilitating mental practice that aligns with their unique learning pace and habits.

### 5.3 Advancing VMI effectiveness measurement with physiological approaches

While VMI has been shown to have multiple benefits in music practice, including enhancements in self-efficacy, motivation, and anxiety management, the methodologies used to assess these benefits remain limited. Traditional approaches include laboratory-based experiments (e.g., Cahn, 2008), subjective self-reports (e.g., Clark and Williamon, 2011), performance evaluations from external judicators (e.g., Lim and Lippman, 1991) and follow-up interviews (Hatfield, 2016). Although these methods could present the effectiveness of mental imagery via both qualitative and quantitative data, they come with inherent limitations. For instance, laboratory experiments are usually constrained by small sample sizes with simplistic tasks and under researchers’ high control. These elements can lead to experimental settings that deviate markedly from participants’ usual practice environments, potentially affecting the results with unexpected biases (Bernardi et al., 2012). Considering the pivotal role that bodily movements play in manifesting musical cognition and expression (Luck and Toiviainen, 2008), the potential of employing wearable technology (e.g., smartwatches, wristbands, and rings) to monitor physiological markers (such as heart rate, skin temperature, and blood pressure), or the use of unintrusive markerless motion capture solutions, merits attention. Such an approach could provide researchers with objective data reflecting the reality of participants’ engagement with VMI within their familiar environments over extended periods and prompt a novel perspective for understanding musical imagery via physi(ologi)cal correlates.

## 6 Conclusion

In this review, we defined what voluntary musical imagery is, why it might support musicianship enhancement, and how it might be applied effectively. Our analysis of current studies has broadened the definition of VMI. We propose that VMI is not merely a mental rehearsal of music but an embodied phenomenon where imagery and bodily processes are deeply interconnected. This view is supported by evidence of neural equivalence, showing overlapping and correlated brain activity during the imagination, perception, and physical execution of music. Furthermore, empirical studies that have applied VMI in deliberate practice, self-regulated learning (SRL), and mental practice have demonstrated its utility in enhancing musical practice both mentally and physically. However, limitations within the current body of research on VMI have also been highlighted, such as the lack of empirical data between embodied musical experience and the formation of VMI, the dearth of musicians’ personalized VMI application, and the need for novel objective instruments for measuring VMI’s effectiveness.

Furthermore, this review offers critical insights for music educators, performers, and researchers into the application of VMI. Specifically, educators are encouraged to apply VMI to their teaching, promoting VMI as a complementary technique to physical practice for enhancing learning efficacy. Performers, meanwhile, can leverage VMI for mental preparation, potentially reducing anxiety and improving performance quality. For researchers, the call to action involves adopting more rigorous, unified methods to further unravel VMI’s complexities and its influence on musicianship.

Moreover, VMI’s utility is not confined to formally trained musicians. It offers potential benefits to individuals across various levels of musical engagement, including hobbyists and self-taught musicians. Beyond the music domain, VMI’s applications may extend to other performance-driven fields, such as sports, surgery, and business, where mental rehearsal and imagery are crucial for enhancing skill development and performance outcomes (Maring, 1990; Conlin et al., 2016; Clark and Williamon, 2011).

In conclusion, this literature review contributes analysis to the discourse on Voluntary Musical Imagery (VMI), advocating for its integrated role in enhancing music education and performance.
